# Farmers’ knowledge, perceptions, and practices on animal trypanosomosis and the tsetse fly vector: A cross-sectional study around Kenya’s Arabuko-Sokoke Forest Reserve at the livestock-wildlife interface

**DOI:** 10.12688/openresafrica.13397.1

**Published:** 2022-06-06

**Authors:** Erick K Serem, Joel L Bargul, Moses M Ngari, Osman A Abdullahi, David M Mburu

**Affiliations:** 1 Department of Public Health, School of Health and Human Sciences, Pwani University, Kilifi, P.O Box 195-80108 Kilifi, Kenya; 2 Pwani University Bioscience Research Centre (PUBReC), Pwani University, Kilifi, P.O Box 195-80108, Kenya; 3 Department of Biochemistry, Jomo Kenyatta University of Agriculture and Technology (JKUAT), Nairobi, P.O Box 62000-00200, Kenya; 4 Animal Health Theme, International Centre of Insect Physiology and Ecology (icipe), Nairobi, P.O Box 30772-00100, Kenya; 5 KEMRI/Wellcome Trust Research Programme, Clinical Trials Facility, Kilifi, P.O Box 230-80108, Kenya; 6 Department of Biological Sciences, Pwani University, Kilifi, P.O Box 195-80108, Kenya

**Keywords:** animal trypanosomosis, tsetse flies, wildlife–livestock interface, small-scale farmers, Arabuko-Sokoke Forest Reserve, Kenya

## Abstract

**Background:** Animal African trypanosomosis (AAT) is a veterinary disease caused by trypanosomes transmitted cyclically by tsetse flies. AAT causes huge agricultural losses in sub-Saharan Africa. Both tsetse flies and trypanosomosis (T&T) are endemic in the study area inhabited by smallholder livestock farmers at the livestock-wildlife interface around Arabuko-Sokoke Forest Reserve (ASFR) in Kilifi County on the Kenyan coast. We assessed farmers’ knowledge, perceptions and control practices towards T&T.

**Methods:** A cross-sectional study was conducted during November and December 2017 to collect data from 404 randomly selected cattle-rearing households using a structured questionnaire. Descriptive statistics were used to determine farmers’ knowledge, perceptions, and control practices towards T&T. Demographic factors associated with knowledge of T&T were assessed using a logistic regression model.

**Results:** Participants consisted of 53% female, 77% married, 30% elderly (>55 years), and the majority (81%) had attained primary education or below. Most small-scale farmers (98%) knew the tsetse fly by its local name, and 76% could describe the morphology of the adult tsetse fly by size in comparison to the housefly’s ( *Musca domestica*). Only 16% of the farmers knew tsetse flies as vectors of livestock diseases. Higher chances of adequate knowledge on T&T were associated with the participants’ (i) age of 15–24 years (aOR 2.88 (95% CI 1.10–7.52), (ii) level of education including secondary (aOR 2.46 (95% CI 1.43–4.24)) and tertiary (aOR 3.80 (95% CI 1.54–9.37)), and (iii) employment status: self-employed farmers (aOR 6.54 (95% CI 4.36–9.80)).

**Conclusions:** Our findings suggest that small-scale farmers around ASFR have limited knowledge of T&T. It is envisaged that efforts geared towards training of the farmers would bridge this knowledge gap and sharpen the perceptions and disease control tactics to contribute to the prevention and control of T&T.

## Introduction

African trypanosomes ( *Trypanosoma*) are flagellated parasitic protozoa that are cyclically transmitted by tsetse flies ( *Glossina* spp.), causing neglected tropical disease, human African trypanosomiasis (HAT) and animal African trypanosomosis (AAT) ( Capewell *et al.*, 2015). Tsetse flies, which are obligate blood feeders, are found in 38 sub-Saharan African (SSA) countries, including Kenya ( FAO, 2016). However, mechanical transmission of animal trypanosomes has been documented to occur through other dipterans including tabanids and *Stomoxys* spp. The latter may explain the occurrence of AAT outside the continental range of tsetse flies. For instance, *Trypanosoma evansi* and *T. vivax* infections were reported in horses, mules, camels, and cattle found in Asia and South America ( Desquesnes & Dia, 2003; Jones & Dávila, 2001; Mihok *et al.*, 1995).

AAT is caused by parasitic *T. brucei brucei*, *T. evansi*, *T. vivax*, *T. congolense*, and *T. equiperdum* ( Auty *et al.*, 2012; Giordani *et al.*, 2016) and infects almost all domestic animals including cattle, sheep, goats, pigs, horses, camels, and dogs ( Desquesnes & Dia, 2004; Desquesnes *et al.*, 2013; Matete, 2003; Singh *et al.*, 1993). Notably, wildlife act as key reservoirs of many pathogens, which are transmitted to humans and livestock by hematophagous biting flies particularly at the livestock-wildlife interface ( Ebhodaghe *et al.*, 2021). Previous studies have reported trypanosomes in wild animals, including warthog *Phacochoenis aethiopicus,* African buffalo *Syncerus caffer*, giraffe *Giraffa camelopardalis*, African savannah elephant *Loxodonta africana*, and hippopotamus *Hippopotamus amphibious* ( Anderson *et al.*, 2011; Clausen *et al.*, 1998; Muturi *et al.*, 2011; Snow *et al.*, 1988). Recent studies on bloodmeal analyses in field-collected tsetse flies from the Kenya’s Shimba Hills National Reserve found that the warthog *Phacochoerus africanus* is the major cryptic reservoir of animal trypanosomes causing diseases in cattle kept at the wildlife-livestock interface ( Ebhodaghe *et al.*, 2021)

AAT is a serious veterinary disease that causes huge socio-economic burden in SSA ( Shaw *et al.*, 2014). The disease exacerbates poverty and contributes to food insecurity through (1) loss of draught power due to weakening of infected animals, (2) productivity losses (reduced milk and meat), (3) additional animal treatment costs, and (4) high morbidity and mortality in livestock ( Fasina *et al.*, 2021; Steverding, 2008). Occurrence of AAT, which is endemic in 38 of the 47 counties in Kenya ( Kenya Ministry of livestock development, 2011), together with its tsetse fly vector, presents a major constraint to livestock production, thus hindering economic development among the smallholder livestock farmers. The benefits that could accrue from tsetse and trypanosomosis prevention and control could greatly improve the living standards of the pastoral communities inhabiting disease endemic regions bordering the wildlife reserves, for instance Arabuko-Sokoke Forest Reserve (ASFR), Kenya.

ASFR is the largest and most intact coastal forest covering an area of 420 km ^2^ ( www.kenyaforestservice.org). This forest is inhabited by four species out of the 31 tsetse species and subspecies described broadly classified into three groups: the Morsitans (Savannah), the Fusca (Forest) and Palpalis (Riverine). ASFR is inhabited by two species of tsetse flies of the Savannah ( *G. pallidipes*, and *G. austeni*) and two of the Forest groups namely *G*. *brevipalpis* and *G. longipennis,* ( Ngari *et al.*, 2020). Human encroachment of formerly forested areas bordering the ASFR, in search of arable land and pastures for their livestock, promotes transmission of tsetse-transmitted diseases of veterinary and zoonotic importance through habitat sharing ( Rutto *et al.*, 2013).

Knowledge and perception towards vector-borne diseases and their transmitting vectors among the livestock keepers in endemic areas is crucial for disease prevention and control at household level ( Tshimungu *et al.*, 2008). However, acquired knowledge, perceptions, prevention and control practices among the community towards the vector and the disease are not well understood. We conducted a cross-sectional survey around the ASFR to determine farmers’ knowledge, perceptions, preventive and control practices towards tsetse fly and trypanosomosis (T&T).

## Methods

### Study design and study area

**Figure 1.**
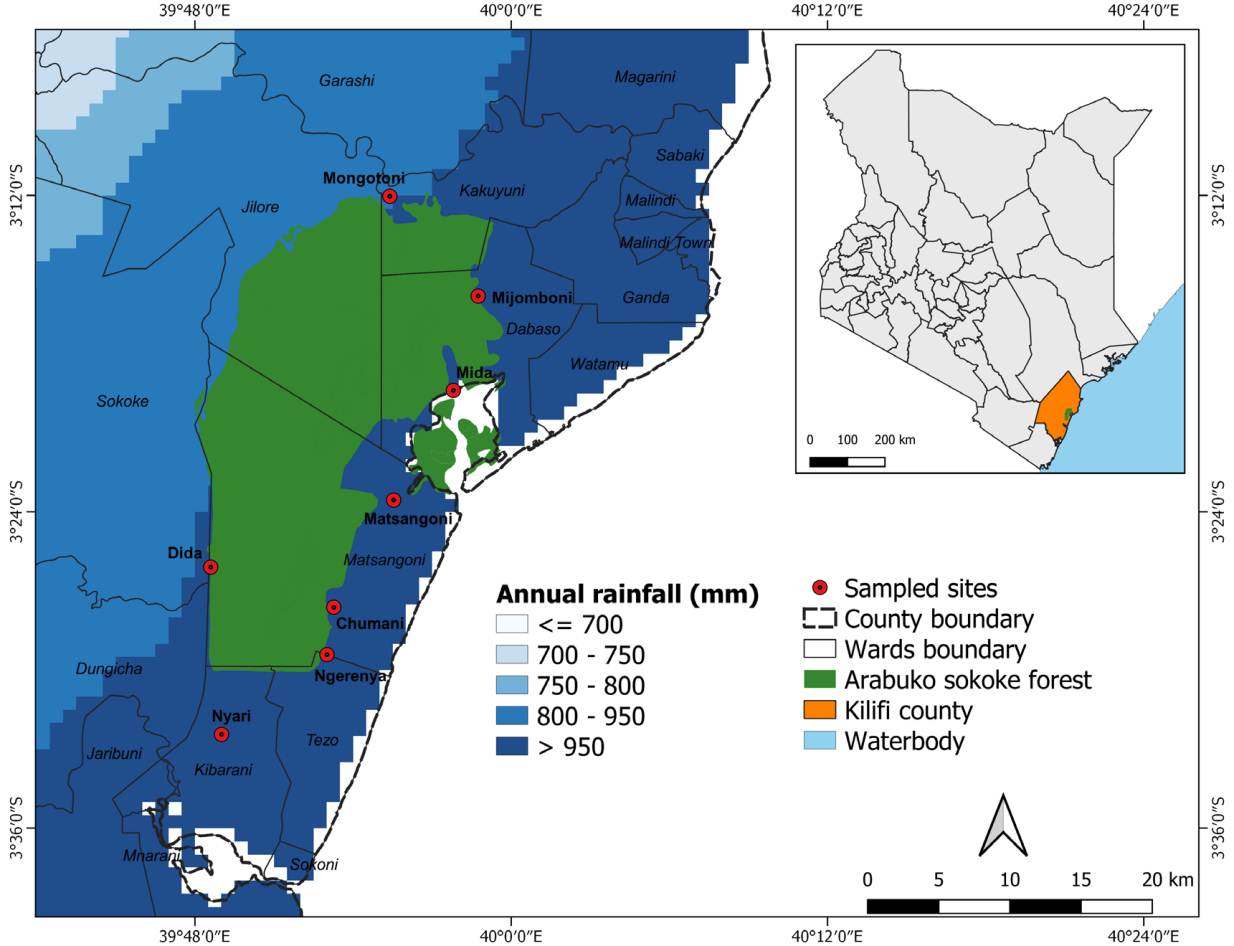
A map of Kenya showing the sampling locations surrounding Arabuko-Sokoke Forest Reserve in Kilifi County. The figure was generated using open-source software QGIS version 3.18 (RRID:SCR_018507). The annual rainfall data was obtained from Climate Hazards group Infrared Precipitation with Stations ( CHIRPS).

A cross-sectional study was conducted between November and December 2017 from three sub-counties of Kilifi County, namely Ganze, Kilifi North, and Malindi. The study was conducted at the wildlife-livestock interface around Arabuko-Sokoke Forest Reserve (ASFR) in Kilifi County, Kenya ( Figure 1). Kilifi County lies between latitude 2 ^° ^20 ^'^ and 4 ^° ^0 ^' ^South, and between longitude 39 ^°^ 05 ^'^ and 40 ^°^ 14 ^'^ East. The County borders Kwale County to the southwest, Taita Taveta County to the west, Tana River County to the north, Mombasa County to the south, and Indian Ocean to the east. Kilifi County covers an area of about 12,609.7 km ^2^ with an estimated human population size of 1,453,787 people in 2019 ( KNBS, 2019). The County has a total of seven sub-counties, namely Kilifi North, Kilifi South, Ganze, Malindi, Magarini, Rabai, and Kaloleni.

Our study area neighbouring the ASFR is mainly inhabited by the Giriama subtribe of the Mijikenda. Most people derive part of their livelihoods from small scale crops and livestock farming as well as fishing ( MoALF, 2016). The livestock reared are of indigenous breeds including Coastal Zebu and Orma Boran, the exotic breeds including Friesian and Ayrshire, goats, sheep, poultry and beekeeping ( KMALDFC, 2019).

### Weather conditions

The average temperature in areas around ASFR where tsetse flies are endemic is 26.5 °C (24–32 °C; March and June are the warmest and the coldest months of the year respectively). About 900 to 1200 mm of precipitation falls annually. February and May are the driest and most wet months with a precipitation of ~21 mm and 173 mm respectively. The average relative humidity (R.H.) is 74% per annum with a maximum R.H. of 80% in May, and a minimum of 70% in February. The weather during the long rains between April and July is conducive for the replication of tsetse flies as they have a temperature limit between 26 °C and 32 °C and the vector can survive at an R.H. range of 40–76% ( Wamwiri *et al.*, 2013). The typical habitat for the Morsitans group of tsetse flies is open woodland and woodland savanna ecosystem that is mainly composed of *Brachystegia* ( Krinsky, 2002). ASFR has three types of forest cover including mixed forest comprising *Brachystegia* and *Cynometra* tree species preferred by *G. austeni*, *Brachystegia* woodland preferred by *G. pallidipes* and *Cynometra* forest, which is preferred by *G. brevipalpis* and *G. longipennis* ( Cecchi *et al.*, 2008; De Beer *et al.*, 2021).

### Study participants and the inclusion criteria

The study targeted households with participants aged ≥15 years, lived ~ 5 km from the border of ASFR, and kept livestock including cattle, sheep, and goats. We obtained a list of eligible households from the local administration (chiefs’) offices from which a sample size of 404 participants was obtained as described below.

### Sample size determination

We calculated our sample size using an estimated prevalence of 50% to compute the sample size at a level of significance of 0.05 and precision of ± 0.05, which resulted in a sample size of 385 (i.e. 1.96 ^2 ^× 0.5(1-0.5)/0.05 ^2^ = 385) ( Daniel, 1999). We adjusted this sample size of 385 upwards by 5%, thus giving a final sample size of 404 (i.e. 1.05 × 385 = 404.25) as a contingency plan to cover for the unexpected cases of withdrawal from the study by the selected participants.

### Ethical considerations

Ethical approval was obtained from the Pwani University Ethics Review Committee (approval number: ERC/PhD/015/2016). Permission to conduct the study was granted by the area Chief, village elders, and livestock farmers after a series of sensitization and stakeholder meetings to engage the local community on the information about the proposed research. Subsequently, both verbal and written informed consent ( Serem *et al.*, 2022) were obtained from the eligible participants, and from the parents of those participants aged below 18 years (but not less than 15), prior to administration of the questionnaires.

### Data collection

Eight sampling sites were randomly selected from 32 eligible locations that surrounded the ASFR as described in the inclusion criteria and these included Dida, Nyari, Chumani, Matsangoni, Mida-Majaoni, Mijomboni, Mongotini, and Ngerenya ( Figure 1). Households for interviews (n =404) were chosen at random in each of the eight locations via the lottery method by considering the proportion of eligible participants in each location.

The household heads were interviewed and in case they were not available we engaged the most knowledgeable member of the household, who actively engaged in livestock herding duties with experience of grazing animals at the border of ASFR.

The data was collected using structured questionnaires ( Serem *et al.*, 2022) adapted from Mwaseba & Kigoda (2017) from the consenting participants to determine their knowledge, perceptions and practices towards control of livestock animal trypanosomosis disease and its vector, the tsetse fly. The structured questionnaire as a tool consisted of four sections, namely demographics, knowledge, perceptions and practices. The tool included both closed and open-ended questions. Trained field assistants administered the questionnaires in Giriama language understood by the respondents.

### Statistical analysis

Analysis of data was done using STATA software version 17.1 (RRID:SCR_012763). Data on farmer’s knowledge, perceptions, and practices towards tsetse flies and animal trypanosomosis were analyzed using descriptive statistics. Logistic regression was used to analyse the demographic factors associated with knowledge towards T&T. The following three criteria were used in scoring the knowledge of the participant (i.e. the dependent variable); the ability of the respondent to (1) state the local name of tsetse fly in a language spoken by the community, (2) provide accurate description of the tsetse fly, and (3) identify livestock diseases transmitted by tsetse flies.

Backwards stepwise binary logistic regression analysis was used to determine the demographic factors associated with the knowledge of T&T by retaining variables with P <0.1 in the multivariable model. The data were reported using both crude and adjusted odds ratios and their respective 95% confidence intervals. We assessed the goodness of fit and performance of multivariable regression model using the Hosmer–Lemeshow test and area under receiver operating characteristics curve (AUC). The sampling locations were organized into clusters and robust standard errors were computed to account for the clustering effect in the multivariable regression model.

## Results

### Demographic characteristics

Of the 404 participants, 53% (214/404) were female, about one third consisted of elderly aged ≥55 years (30%; 123/404), 12% (47/404) were single and the rest were either married, divorced, separated, or widowed. About 33% of the participants lacked formal education (134/404), and the rest attained the following levels of education: primary 47.5% (192/404), secondary 15% (61/404), and tertiary level 4.5% (16/404). Furthermore, 10% of the participants were in formal employment, while the rest were either self-employed or unemployed. The number of selected participants from each of the eight sampling sites is summarized in Table 1.

**Table 1.** The number and site-specific distribution of randomly selected livestock-keeping respondents living near the border of Arabuko-Sokoke Forest Reserve (ASFR).

S/No	Sampling locations bordering ASFR	Sample distribution; Percentage % ( *n/404)
1	Matsangoni	12 (47/404)
2	Dida	13 (51/404)
3	Chumani	13 (53/404)
4	Mijomboni	13 (53/404)
5	Mida	13 (54/404)
6	Ngerenya	11 (43/404)
7	Kakuyuni	13 (53/404)
8	Nyari	12 (50/404)

*n: represents the number of respondents from each sampling site.

### Knowledge of the livestock diseases

**Figure 2.**
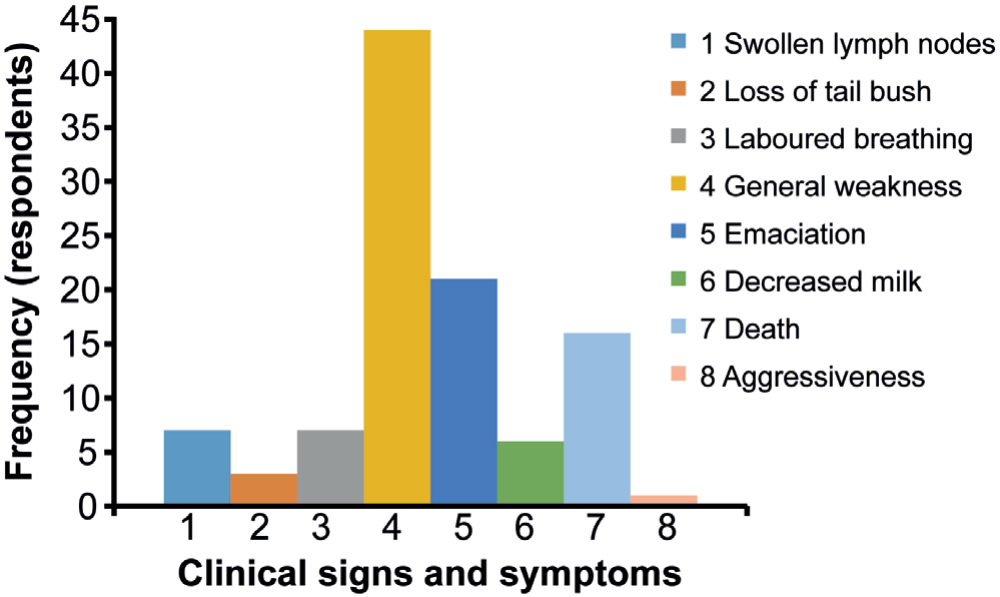
Health problems in livestock that were associated with tsetse-transmitted diseases. Data was collected from the respondents (n = 65) who had knowledge of tsetse flies as transmitters of diseases.

Nearly all participants (98%; 397/404) reported to have knowledge of tsetse flies and 87.4% (353/404) of them knew the local name of the tsetse *Imbu* in Giriama; *Mbung’o* (2.5%) in Swahili, and the Mijikenda subtribe of Chonyi call it *Chibu* (8.2%). The majority of the participants (76%) could only describe tsetse morphology based on size i.e., approximately the size of a housefly, while 161 participants (40%) reported their observation of the tsetse to have long mouthparts for blood-feeding. A further 23% of the respondents described tsetse flies by their body colour to be ‘brown-grey’ to ‘brown-dark’. Information on where livestock and tsetse flies most likely interact, the abundance of tsetse flies, challenges that tsetse flies pose to livestock, and the diseases transmitted by these vectors are summarized in Table 2. A small fraction of the respondents, 16% (65/404), were aware that tsetse flies are vectors of livestock diseases, from which only 6.1% (4/65) ably described the link between tsetse and trypanosomosis (nagana). About 80% of the respondents who knew that tsetse flies transmit trypanosomosis reported infections in cattle, while 43% reported infections in goats and sheep. Health problems associated with tsetse-transmitted trypanosomosis were reported by the participants ( Figure 2).

**Table 2.** Summary of the participants’ knowledge on tsetse fly, its ecology, seasonal fly densities, and vector-borne transmission of livestock diseases.

Knowledge	Percentage % ( *n/404)
*Where do tsetse flies live?*	
Grassland	18.0 (74/404)
Bottom of valley	2.2 (9/404)
Bush area	84.0 (340/404)
Hilly areas	0.5 (2/404)
*Where do livestock get into close * *contact with tsetse flies?*	
Forest	91.0 (368/404)
Riverine area	1.2 (5/404)
Un-forested areas	7.2 (29/404)
Don’t know	4.0 (16/404)
*Which season of the year has the * *highest numbers of tsetse flies?*	
Rainy season	72.0 (289/404)
Dry season	7.9 (32/404)
Throughout the year	6.9 (28/404)
Don’t know	12.0 (48/404)
*What problems do tsetse flies * *cause to the livestock?*	
Painful bites	81.0 (326/404)
Distract grazing activities	53.0 (216/404)
Transmit disease	16.0 (64/404)
Don’t know	7.9 (32/404)
*Do tsetse flies transmit diseases to * *livestock?*	16.0 (65/404)
*Which livestock disease do tsetse * *flies spread? (N=65)*	
Homa (fever)	78.0 (51/65)
Nagana	6.1 (4/65)
Don’t know the disease	15.0 (10/65)

*n: represents the number of respondents for each category.

Among the respondents, 75% of them had no prior access to any source of information about tsetse and trypanosomosis. However, 12% of the respondents reported to have previously received basic knowledge of tsetse, either through their encounters with these flies in the forest while performing livestock herding duties, or via engagement with their parents. Other secondary sources of information they received were from: school (6.2%), extension officers (3%), NGOs (1.5%), and mass media (0.2%). We also noted that there were differences in the level of knowledge on T&T in the study sites. Kakuyuni had the highest number of participants with adequate knowledge whereas in Mida and Ngerenya, none of the participants had adequate knowledge ( Table 3). Dida had the highest number of participants with tertiary education at 33.33%. 

**Table 3.** The number and site-specific distribution of randomly selected livestock-keeping respondents with adequate knowledge of tsetse flies and trypanosomosis.

S/No.	Sampling locations bordering ASFR	Sample distribution; Percentage % (*n/65)	No formal education (*n/13)	Primary (*n/28)	Secondary (*n/18)	Tertiary (*n/16)
1	Matsangoni	18.46 (12/65)	15.38(2/13)	28.57 (8/28)	5.56 (1/18)	16.67 (1/16)
2	Dida	23.08 (15/65)	23.08 (3/13)	17.86 (5/28)	27.78 (5/18)	33.33 (2/16)
3	Chumani	13.85 (9/65)	0	17.86 (5/28)	16.67 (3/18)	16.67 (1/16)
4	Mijomboni	4.62 (3/65)	7.69 (1/13)	0	5.56 (1/18)	16.67 (1/16)
5	Mida	0 (0/65)	0	0	0	0
6	Ngerenya	0 (0/65)	0	0	0	0
7	Kakuyuni	26.15 (17/65)	46.15 (6/13)	28.57 (8/28)	16.67 (3/18)	0
8	Nyari	13.85 (9/65)	7.69 (1/13)	7.14 (2/28)	27.78 (5/18)	16.67 (1/16)

### Perceptions of farmers

One hundred and nineteen (30%) participants adopted tsetse control techniques in order to prevent livestock infestation by tsetse flies. About 17% and 18% of the respondents felt that the recommended tsetse control measures were easy to use and effective, respectively, while 78% and 79% were not sure about the ease of use and effectiveness of the control measures, respectively. A further 17% of the farmers were hopeful that the control methods were reliable for tsetse control, and only 7.4% of the participants expressed their opinion that the recommended control strategies were affordable. Three hundred and sixteen participants (78%) were neither sure whether the recommended tsetse control methods were easy to deploy nor effective, whereas 320 (79%) were not sure of the affordability of the control methods. Other parameters associated with the perceptions of the participants on the reasons for persistence of tsetse transmitted diseases are shown in Table 4.

**Table 4.** Summary of the participants’ perceptions on the reasons for the persistence of tsetse flies and effects of tsetse flies.

Perceptions	Percentage % (*n/404)
*What are the reasons for persistence of tsetse-transmitted livestock diseases?*	
Lack of knowledge on nagana disease	2.2 (9/404)
Residing near ASFR	8.9 (36/404)
Lack of community animal health workers and other experts to train farmers	0.7 (3/404)
Presence of tsetse flies	3.0 (12/404)
I do not know	14.0 (55/404)
*What are the outcomes of tsetse flies’ infestation on community members?*	
Painful bites	16.0 (64/404)
Disease transmission	18.0 (72/404)
Financial losses	2.7 (11/404)
I do not know	68.0 (273/404)

### Practices towards tsetse control

Among the 30% of the respondents who practiced tsetse control, the main tsetse control methods employed and the perceived reasons for persistence and effects of tsetse-transmitted diseases are summarized in Table 5.

**Table 5.** Practices towards tsetse control.

Practices against the tsetse fly vector	Percentage % ( *n/404)
*Have you tried to prevent tsetse fly flying near animals?*	30.0 (119/404)
*If yes, what ways of tsetse control have you tried before?*	% ( *n/119)
Avoiding tsetse-infested areas	9.2 (11/119)
Burnt herbs near livestock herds to repel tsetse	19.0 (23/119)
Spraying livestock with commercially available insecticides	51.0 (61/119)
Applying used engine oil on the animal’s body	0.8 (1/119)
Using fly traps and targets	0.8 (1/119)
Clearing neighbouring tsetse-infested bush	18.0 (21/119)

*n: represents the number of respondents for each category.

### Association of the demographic factors with the knowledge of tsetse fly vector

Sixty-five participants (16%, 95% CI 13 to 20%) had adequate knowledge of tsetse flies. In the univariate logistic regression models, males were associated with higher odds of adequate tsetse fly knowledge (crude odds ratio of 2.55 (95% CI 1.24–5.24)) than females ( Table 6). Further, in comparison to the participants without formal education, both secondary and tertiary education levels were associated with higher odds of adequate knowledge of tsetse flies: crude odds ratios 3.90 (95% CI 1.47–10.32) and 5.58 (95% CI 2.01–15.50), respectively ( Table 6).

**Table 6.** Association of the demographic factors with the knowledge on tsetse flies.

Demographic characteristics	Adequate knowledge of tsetse fly		Univariate analysis		Multivariable analysis	
	Yes (n = 65)	No (n = 339)	Crude odds Ratios	P-value	Adjusted odds Ratios	P-value
**Sex**						
Male	23(43/190)	77(147/190)	2.55 (1.24–5.24)	0.01	1.96 (0.88–4.35)	0.10
Female	10(22/214)	90(192/214)	Reference		Reference	
**Age (in years)**						
Above 55	13(16/123)	87(107/123)	Reference		Reference	
45 to 54	16(9/55)	84(46/55)	1.31 (0.61–2.82)	0.49	1.18 (0.51–2.74)	0.69
35 to 44	16(15/91)	84(76/91))	1.32 (0.57–3.05)	0.52	1.05 (0.45–2.43)	0.91
25 to 34	17(13/78)	83(65/78)	1.34 (0.59–3.03)	0.49	1.15 (0.51–2.57)	0.73
15 to 24	21(12/57)	79(45/57)	1.78 (0.67–4.74)	0.25	2.88 (1.10–7.52)	0.03
**Marital status**						
Married	16(51/312)	84(261/312)	Reference			
Single	23(11/47)	74(36/47)	1.56 (0.51–4.84)	0.44	*	
Divorced	25(1/4)	75(3/4)	1.71 (0.12–24.2)	0.69	*	
Separated	25(1/4)	75(3/4)	1.71 (0.15–19.5)	0.67	*	
Widowed	3(1/37)	97(36/37)	0.14 (0.02–1.05)	0.06	*	
**Level of formal education**						
No formal education	9.7(13/134)	90(121/134)	Reference		Reference	
Primary	15(28/192)	85(164/192)	1.59 (0.92–2.73)	0.10	1.12 (0.63–1.98)	0.69
Secondary	30(18/61)	70(43/61)	3.90 (1.47–10.3)	0.006	2.46 (1.43–4.24)	0.001
Tertiary	38(6/16)	62(10/16)	5.58 (2.01–15.5)	0.001	3.80 (1.54–9.37)	0.004
**Occupation**						
Self-employed	24(44/184)	76(140/184)	4.37 (1.69–11.3)	0.002	6.54 (4.36–9.80)	<0.001
Unemployed	7(12/178)	93(166/178)	3.91 (1.06–14.4)	0.04	3.06 (0.71–13.2)	0.13
Employed	22(9/41)	78(32/41)	Reference		Reference	

*No results because the variable was not selected using backward stepwise for inclusion in the multivariable model, Crude and adjusted odds ratios are from logistic regression models.

Based on multivariable regression analysis, the participant’s aged between 15 and 24 years were associated with higher odds of adequate understanding of the tsetse (aOR 2.88 (95% CI 1.10–7.52)), relative to the older ones aged above 55 years. Further, attainment of secondary education (aOR 2.46 (95% CI 1.43–4.24)) and tertiary education (aOR 3.80 (95% CI 1.54–9.37)) was associated with higher odds of adequate knowledge of tsetse flies than lack of it (formal education). In addition, self-employment was associated with higher odds of adequate tsetse fly knowledge (aOR 6.54 (95% CI 4.36–9.80)) relative to formal employment ( Table 6).

We tested the effectiveness of our multivariable regression model AUC (95%) and found its predictive performance to be 0.76 (95% CI 0.70–0.81) and, also, the Hosmer–Lemeshow goodness of fit test value for the model was 5.06 and P = 0.75.

## Discussion

In this study conducted around the ASFR in Kenya, we assessed the knowledge, perceptions, and practices of smallholder livestock farmers on tsetse flies and the disease they transmit, animal African trypanosomosis using a structured questionnaire for data collection. Our survey revealed important socio-demographic characteristics of the small-scale farmers living at the wildlife-livestock interphase in ASFR. Three parameters: age, level of education and employment status, were significant demographic characteristics in a logistic regression model which had an association with knowledge of T&T. These observations corroborate results by Laskowski *et al*. (2011) who found that age, sex, level of education and occupation played an important role in the epidemiology of communicable diseases. From this survey, only 19% had secondary level or tertiary level of education. Contrary to other studies where the respondents were predominated by men ( Mwaseba & Kigoda, 2017; Uba *et al.*, 2016), more than half (53%) of the respondents in this study were female and about a third of them were elderly. The higher number of women could be explained by the fact that women dominate livestock keeping in the Giriama community, and the higher number of older people sampled in this study could be due to their preference to live in villages, as opposed to the younger educated generation who prefer to live and work in the major towns of Kilifi and Malindi within the County.

Therefore, the intervention measures for the prevention of AAT would mainly target the elderly, those with low levels of education and the self-employed farmers, who are the primary livestock keepers among the Giriama community surrounding ASFR. Due to the low levels of education among the participants, it would be necessary to package the health education message in a simple way as the level of education may affect the act of reading and subsequent understanding of the information, education and communication (IEC) material. Though sex was not a statistically significant parameter in the regression model, we noted that women were the main “care givers” of livestock in the study area and thus it would be important to prioritize them during health education.

Nearly all participants (98%) reported to have knowledge of tsetse flies and identified them by the local name of the tsetse fly, whereas 77% of the respondents could describe the tsetse based on the size of the adult fly, which is approximately the size of *G. austeni,* which is endemic in this region. However, overall, only 16% of the participants had adequate knowledge on T&T. Inadequate knowledge about a disease poses a major challenge in eradicating or reducing its transmission. The limited knowledge on T&T among the study participants shows the challenge of reducing transmission in this setting. One third of the participants were either elderly or had no formal education suggesting the need for more targeted training in language the farmers can understand using existing systems like the veterinary officers or local administration.

Our study findings are comparable to those of the farmers living near Shimba Hills National Reserve in Kenya where only 23.1% of farmers could link the causal association between tsetse flies and trypanosomosis ( Machila *et al.*, 2003). Another study, in similar setting as ASFR, carried out among participants living near Serengeti National Park in Tanzania, showed that the majority of the respondents could only identify tsetse flies by size and could not distinguish them from other similar dipteran ( Mwaseba & Kigoda, 2017). In a study done among people living in and around Serengeti National Park, the participants, especially those living in the park, had adequate knowledge of trypanosomosis ( Kinung’hi *et al.*, 2006). The reason for the high level of knowledge in the Serengeti may be attributed to the high number of respondents (45%) who had attained secondary education. The latter level is higher in comparison to 15% for those with secondary and post-secondary education (4.5%), those without formal education (33 %) and those with primary level education comprising 47.5% in this study. This also underscores the fact that knowledge levels could change over time ( Bates, 2015) and this needs to be assessed from time to time and the identified gaps filled through health education.

Knowledge is important in the management of diseases and participants who are familiar with the clinical signs of AAT are more likely to seek veterinary assistance because they can identify sick animals early before their body conditions deteriorate ( Seyoum *et al.*, 2013). Most of the respondents were unable to describe the clinical signs of an animal suffering from trypanosomosis. We also noted that though most of the people in formal employment had post-primary education, the self-employed farmers in this study had better knowledge of tsetse flies. This could be due to the fact that most of the employed work away from the villages and are not directly involved in livestock keeping as opposed to the farmers, the majority of which had primary or no formal education. Overall, participants in Kakuyuni had the highest level of knowledge on T&T (26%) compared to other study sites sampled despite only 16% having post primary education. This may be due to the fact that Malindi Sub County, where Kakuyuni is located, has consistently had the highest number of trypanosome cases in the entire Kilifi County (Director Veterinary Services quarterly reports ( Serem *et al.*, 2022)) and probably more farmers have seen many cases of AAT. The other location with higher levels of knowledge was in Dida, Ganze sub-County (23%), where members of the community reported increased numbers of tsetse flies especially when elephants go to the water point at the edge of the forest and it had the highest number of participants with post primary level of education (61%).

Any type of illness is referred to as “homa” by the Giriama, which means “fever”. In this study 78% of those who knew that tsetse flies transmit diseases believed that tsetse fly bites cause “homa” in humans and in livestock. Furthermore, the limited knowledge among the participants on T&T negatively influenced their perception towards prevention and control measures against T&T. About 30% of the participants were aware of the control measures and may have used one or more practices to prevent tsetse flies from biting their animals. However, only a small fraction (17%) of the participants felt that the control measures were reliable and only 7.4% felt that tsetse control strategies were affordable. Though some of the participants knew what should be done to control the flies, they could not implement the control measures due to affordability of the cost. It has, however, been observed that community willingness to contribute resources and participate in control activities is influenced by knowledge of tsetse flies and the control measures towards trypanosomosis ( Sindato *et al.*, 2008). We believe that once the farmers are knowledgeable on T&T, and the potential losses they could incur if they do not implement the control measures, their perceptions and priorities will change.

Among the respondents who implemented tsetse control practices, we observed that some of the farmers dipped or sprayed their livestock, though they were unaware that some acaricides could prevent tsetse flies from contacting and biting their livestock. While this study did not look into the types of acaricides used by participants, other studies in Uganda found that farmers may use acaricides to which tsetse flies are resistant but only effective against ticks ( Bardosh *et al.*, 2013) thus jeopardizing tsetse control. Some of the participants tried to burn some herbal plants near the herd in order to repel tsetse flies while others cleared bushes around the herd holding area so as to prevent resting sites for the tsetse flies. Though smoke from wood ( *Colophospermum mopane*) and cow dung has been shown to repel tsetse flies ( Torr *et al.*, 2011), it would also be important to carry out more studies on the herbs used by farmers in the study area to assess their real potential in repelling tsetse flies.

In a study by Kumaran *et al.* (2018) it was found that disease knowledge does not always correlate with control practice, and participants with high knowledge levels may not practice preventive measures. Understanding of how AAT affects cattle owners and how they deal with the disease is vital to development of effective, locally tailored disease control programmes ( Holt *et al.*, 2016) which may include changing their perception. Our results corroborate the observation by Alobuia *et al.* (2015) that low level of education among communities negatively influences preventive and control practices. Moreover, low level of education leads to community vulnerability and may require a lot of support to integrate them into preventive and control programmes to mitigate diseases ( Kumaran *et al.*, 2018). Improving the knowledge levels of the small-scale livestock farmers near ASFR could be done with the support of extension officers incorporating the members of the community. Such collaborations could ensure sustainability of the intervention programmes ( Sindato *et al.*, 2008). The acquired knowledge would influence the perception and downstream prevention and control practices of T&T for effective community engagement in limiting the spread of the AAT. 

The knowledge, perceptions and preventive practices surveys, such as in the current study, could help to identify gaps in community participation in prevention and control measures towards tsetse and trypanosomosis. This could make it easier for T&T management at community level, which could contribute towards sustainability of disease control programmes. However, this study relied solely on data collected by interviewing the farmers, which could be skewed due to reporting bias as described by Bandura’s self-efficacy theory ( Bandura, 1986). In addition, knowledge levels may change over time considering the current advances in technology. With the spread of smart phones, many members in the community own phones and may have access to new information from Internet sources, therefore there is need to conduct fresh surveys to validate data from previous studies from time to time.

The key findings of this study show a knowledge gap on tsetse flies and trypanosomosis among the majority of smallholder livestock farmers around the ASFR in Kilifi County, particularly among women and the elderly who make up the majority of herders in the community. Therefore, training of farmers should be considered as a way to harness the much needed human resource by involving the community members in sustainable deployment of tsetse and trypanosomosis control programmes. Our findings provide key baseline information to guide further studies, for instance those focused on disease control.

## Data availability

### Underlying data

Open Science Framework: Underlying data for ‘Farmers’ knowledge, perceptions, and practices on animal trypanosomosis and the tsetse fly vector: A cross-sectional study around Kenya’s Arabuko-Sokoke Forest Reserve at the livestock-wildlife interface’. https://doi.org/10.17605/OSF.IO/MQFKC ( Serem *et al.*, 2022).

This project contains the following underlying data:

- Data file 1: Underlying data.csv
- Data file 2: Clinical cases of trypanosomosis.xlsx
- Data file 3: Statistical analysis.do
- Supplementary file: Data dictionary.xlsx

### Extended data

Open Science Framework: Extended data for ‘Farmers’ knowledge, perceptions, and practices on animal trypanosomosis and the tsetse fly vector: A cross-sectional study around Kenya’s Arabuko-Sokoke Forest Reserve at the livestock-wildlife interface’. https://doi.org/10.17605/OSF.IO/MQFKC ( Serem *et al.*, 2022).

This project contains the following extended data:

- Supplementary file: Consent form and questionnaire.docx

Data are available under the terms of the Creative Commons Zero “No rights reserved” data waiver (CC0 1.0 Public domain dedication).

### Consent

Written informed consent for publication of the participants’ details was obtained from the participants.

## References

[ref-1] Alobuia WM Missikpode C Aung M : Knowledge, Attitude, and Practices Regarding Vector-borne Diseases in Western Jamaica. *Ann Glob Health.* 2015;81(5):654–663. 10.1016/j.aogh.2015.08.013 27036722 PMC4818946

[ref-2] Anderson NE Mubanga J Fevre EM : Characterisation of the wildlife reservoir community for human and animal trypanosomiasis in the Luangwa Valley, Zambia. *PLoS Negl Trop Dis.* 2011;5(6):e1211. 10.1371/journal.pntd.0001211 21713019 PMC3119639

[ref-3] Auty H Anderson NE Picozzi K : Trypanosome Diversity in Wildlife Species from the Serengeti and Luangwa Valley Ecosystems. *PLoS Negl Trop Dis.* 2012;6(10):e1828. 10.1371/journal.pntd.0001828 23094115 PMC3475651

[ref-4] Bandura A : The Explanatory and Predictive Scope of Self-Efficacy Theory. *Journal of Social and Clinical Psychology.* 1986;4(3):359–373. 10.1521/jscp.1986.4.3.359

[ref-5] Bardosh K Waiswa C Welburn SC : Conflict of interest: Use of pyrethroids and amidines against tsetse and ticks in zoonotic sleeping sickness endemic areas of Uganda. *Parasit Vectors.* 2013;6(1):204. 10.1186/1756-3305-6-204 23841963 PMC3711891

[ref-6] Bates W : Teaching in a Digital Age. *Quarterly Review of Distance Education.* 2015;16(4):99.

[ref-7] Capewell P Cooper A Clucas C : A co-evolutionary arms race: trypanosomes shaping the human genome, humans shaping the trypanosome genome. *Parasitology.* 2015;142 Suppl 1(Suppl 1):108–119. 10.1017/S0031182014000602 25656360 PMC4413828

[ref-8] Cecchi G Mattioli RC Slingenbergh J : Tsetse fly habitat and land cover: an analysis at continental level.In *Standardising land cover mapping for tsetse and trypanosomiasis control.*FOOD AND AGRICULTURE ORGANIZATION OF THE UNITED NATIONS.2008;1–17.

[ref-9] Clausen PH Adeyemi I Bauer B : Host preferences of tsetse (Diptera: Glossinidae) based on bloodmeal identifications. *Med Vet Entomol.* 1998;12(2):169–180. 10.1046/j.1365-2915.1998.00097.x 9622371

[ref-10] Daniel WW : Biostatistics: A foundation for analysis in the health sciences.(7th ed.). John Wiley & Sons, Inc., Hoboken.1999. Reference Source

[ref-11] De Beer CJ Dicko AH Ntshangase J : A distribution model for Glossina brevipalpis and Glossina Austeni in southern Mozambique, Eswatini and South Africa for enhanced area-wide integrated pest management approaches. *PLoS Negl Trop Dis.* 2021;15(11):e0009989. 10.1371/journal.pntd.0009989 34843478 PMC8659649

[ref-12] Desquesnes M Dia ML : Mechanical transmission of Trypanosoma congolense in cattle by the African tabanid Atylotus agrestis. *Exp Parasitol.* 2003;105(3–4):226–231. 10.1016/j.exppara.2003.12.014 14990316

[ref-13] Desquesnes M Dia ML : Mechanical transmission of Trypanosoma vivax in cattle by the African tabanid Atylotus fuscipes. *Vet Parasitol.* 2004;119(1):9–19. 10.1016/j.vetpar.2003.10.015 15036572

[ref-14] Desquesnes M Holzmuller P Lai DH : Trypanosoma evansi and surra: A review and perspectives on origin, history, distribution, taxonomy, morphology, hosts, and pathogenic effects. *Biomed Res Int.* 2013;2013:194176. 10.1155/2013/194176 24024184 PMC3760267

[ref-15] Ebhodaghe FI Okal MN Kalayou S : Tsetse bloodmeal analyses incriminate the common warthog *phacochoerus africanus* as an important cryptic host of animal trypanosomes in smallholder cattle farming communities in shimba hills, Kenya. *Pathogens.* 2021;10(11):1501. 10.3390/pathogens10111501 34832656 PMC8623152

[ref-16] FAO: Tsetse and Trypanosomosis Information.(J. Dargie (Ed.); FOOD AND AGRICULTURE ORGANIZATION OF THE UNITED NATIONS.2016;38.

[ref-17] Fasina FO Fasanmi OG Makonnen YJ : The one health landscape in Sub-Saharan African countries. *One Health.* 2021;13:100325. 10.1016/j.onehlt.2021.100325 34584927 PMC8455361

[ref-18] Giordani F Morrison LJ Rowan TG : The animal trypanosomiases and their chemotherapy: a review. *Parasitology.* 2016;143(14):1862–1889. 10.1017/S0031182016001268 27719692 PMC5142301

[ref-19] Holt HR Selby R Mumba C : Assessment of animal African trypanosomiasis (AAT) vulnerability in cattle-owning communities of sub-Saharan Africa. *Parasit Vectors.* 2016;9(1):53. 10.1186/s13071-016-1336-5 26825496 PMC4733274

[ref-20] Jones TW Dávila AMR : Trypanosoma vivax--out of Africa. *Trends Parasitol.* 2001;17(2):99–101. 10.1016/s1471-4922(00)01777-3 11228017

[ref-21] Kenya Ministry of livestock development: Strategy for tsetse and trypanosomiasis eradication in Kenya 2011 –2021. 2011.

[ref-22] Kinung’hi SM Malele II Kibona SN : Knowledge, attitudes and practices on tsetse and sleeping sickness among communities living in and around Serengeti National Park, Tanzania. *Tanzan Health Res Bull.* 2006;8(3):168–172. 10.4314/thrb.v8i3.45115 18254509

[ref-23] KMALDFC: Kenya Livestock Breeds Catalogue. 2019.

[ref-24] KNBS: 2019 Kenya Population and Housing Census Volume 1: Population by County and Sub-County.In: *2019 Kenya Population and Housing Census.* 2019;I. Reference Source

[ref-25] Krinsky WL : Tsetse flies (Glossinidae).In: *Medical and Veterinary Entomology.* Academic Press,2002;303–316. 10.1016/B978-012510451-7/50017-7

[ref-26] Kumaran E Doum D Keo V : Dengue knowledge, attitudes and practices and their impact on community-based vector control in rural Cambodia. *PLoS Negl Trop Dis.* 2018;12(2):e0006268. 10.1371/journal.pntd.0006268 29451879 PMC5833285

[ref-27] Laskowski M Mostaço-Guidolin LC Greer AL : The impact of demographic variables on disease spread: Influenza in remote communities. *Sci Rep.* 2011;1(i):105. 10.1038/srep00105

[ref-28] Machila N Wanyangu SW McDermott J : Cattle owners' perceptions of African bovine trypanosomiasis and its control in Busia and Kwale Districts of Kenya. *Acta Trop.* 2003;86(1):25–34. 10.1016/s0001-706x(02)00288-7 12711100

[ref-29] Matete GO : Occurrence, clinical manifestation and the epidemiological implications of naturally occurring canine trypanosomosis in western Kenya. *Onderstepoort J Vet Res.* 2003;70(4):317–323. 10.4102/ojvr.v70i4.296 14971734

[ref-30] Mihok S Maramba O Munyoki E : Mechanical transmission of Trypanosoma spp. by African Stomoxyinae (Diptera: Muscidae). *Trop Med Parasitol.* 1995;46(2):103–105. 8525279

[ref-31] MoALF: Climate Risk Profile for Kilifi County. Kenya County Climate Risk Profile Series.2016. Reference Source

[ref-32] Muturi CN Ouma JO Malele II : Tracking the feeding patterns of tsetse flies ( *glossina* genus) by analysis of bloodmeals using mitochondrial cytochromes genes. *PLoS One.* 2011;6(2):e17284. 10.1371/journal.pone.0017284 21386971 PMC3046180

[ref-33] Mwaseba DL Kigoda KJ : Knowledge, attitude, and practices about tsetse control among communities neighbouring Serengeti National Park, Tanzania. *Heliyon.* 2017;3(6):e00324. 10.1016/j.heliyon.2017.e00324 28664191 PMC5480271

[ref-34] Ngari NN Gamba DO Olet PA : Developing a national atlas to support the progressive control of tsetse-transmitted animal trypanosomosis in Kenya. *Parasit Vectors.* 2020;13(1):286. 10.1186/s13071-020-04156-5 32503681 PMC7275614

[ref-35] Rutto JJ Osano O Thuranira EG : Socio-Economic and Cultural Determinants of Human African Trypanosomiasis at the Kenya - Uganda Transboundary. *PLoS Negl Trop Dis.* 2013;7(4):e2186. 10.1371/journal.pntd.0002186 23638206 PMC3636132

[ref-36] Serem EK : Knowledge, perceptions and practices of T&T.2022. 10.17605/OSF.IO/MCYWN

[ref-37] Seyoum Z Terefe G Ashenafi H : Farmers' perception of impacts of bovine trypanosomosis and tsetse fly in selected districts in Baro-Akobo and Gojeb river basins, Southwestern Ethiopia. *BMC Vet Res.* 2013;9(1):214. 10.1186/1746-6148-9-214 24139090 PMC4015653

[ref-38] Shaw AP Cecchi G Wint GR : Mapping the economic benefits to livestock keepers from intervening against bovine trypanosomosis in Eastern Africa. *Prev Vet Med.* 2014;113(2):197–210. 10.1016/j.prevetmed.2013.10.024 24275205

[ref-39] Sindato C Kimbita EN Kibona SN : Factors influencing individual and community participation in the control of tsetse flies and human African trypanosomiasis in Urambo District, Tanzania. *Tanzan J Health Res.* 2008;10(1):20–27. 10.4314/thrb.v10i1.14337 18680961

[ref-40] Singh B Kalra IS Gupta MP : *Trypanosoma evansi* infection in dogs: seasonal prevalence and chemotherapy. *Vet Parasitol.* 1993;50(1–2):137–141. 10.1016/0304-4017(93)90014-e 8291188

[ref-41] Snow WF Tarimo SA Staak C : The feeding habits of the tsetse, Glossina pallidipes Austen on the south Kenya coast, in the context of its host range and trypanosome infection rates in other parts of East Africa. *Acta Trop.* 1988;45(4):339–349. 10.5169/seals-314092 2907261

[ref-42] Steverding D : The history of African trypanosomiasis. *Parasit Vectors.* 2008;1(1):3. 10.1186/1756-3305-1-3 18275594 PMC2270819

[ref-43] Torr SJ Mangwiro TN Hall DR : Shoo fly, don't bother me! Efficacy of traditional methods of protecting cattle from tsetse. *Med Vet Entomol.* 2011;25(2):192–201. 10.1111/j.1365-2915.2010.00942.x 21276027

[ref-44] Tshimungu K Kalambayi BB Kiyombo M : Knowledge, behaviours, practices and beliefs regarding human African trypanosomiasis (HAT) among inhabitants of Kinshasa (Democratic Republic of Congo). *Sante.* 2008;18(3):141–147. 10.1684/san.2008.0119 19359235

[ref-45] Uba BV Aliyu A Abubakar A : Knowledge and prevalence of human african trypanosomiasis among residents of kachia grazing reserve, Kachia local government area, Kaduna state, Nigeria, 2012. *Pan Afr Med J.* 2016;23:89. 10.11604/pamj.2016.23.89.7999 27222686 PMC4867183

[ref-46] Wamwiri FN Alam U Thande PC : *Wolbachia, Sodalis* and trypanosome co-infections in natural populations of *Glossina austeni* and *Glossina pallidipes*. *Parasit Vectors.* 2013;6(1):232. 10.1186/1756-3305-6-232 23924682 PMC3751944

