# Supplementary material for: Farmers’ knowledge, perceptions, and practices on animal trypanosomosis and the tsetse fly vector: A cross-sectional study around Kenya’s Arabuko-Sokoke Forest Reserve at the livestock-wildlife interface
Source: Open Res Afr. 2022 Jun 6;5:22. [Version 1] doi: 10.12688/openresafrica.13397.1 (PMC10439355; doi:10.12688/openresafrica.13397.1)
Supplement: Peer review history

# Peer review: Reviewer response for version 1

**DOI:** 10.21956/openresafrica.14545.r30091

**Published:** 2023-08-18

**Authors:** Mumba Chisoni

**Affiliations:** 1 University of Zambia, Lusaka, Zambia, South Africa

The paper presents a very important topic towards developing AAT control policy especially in developing countries.

 The paper is suitable for indexing, subject to minor corrections which I highlight in the review:

- Abstract - Replace "veterinary disease" with a "disease of animals". Do the same to other parts.
- Introduction - The second last paragraph reads like a description of the study area. As such, it should go to the methodology section under the subheading, "Description of study the site".
- Methodology - Why was the study conducted at ASFR and not any other forest? Please provide a justification backed by a reference. Is the estimated prevalence of AAT 50% realistic? I think the authors are mistaking proportion with prevalence. The authors used 50% proportion and not the prevalence of AAT in ASFR.
- What were the ethical issues in this study? How did the authors address them? Include this under ethical considerations other than just writing a statement of where ethical approval was sought.
- Results - The results are presented in a logical manner, and statistics were well explained.
- Discussion - What lessons do you learn from this study? What are the implications of the study on disease control policy?

Is the work clearly and accurately presented and does it cite the current literature?

Yes

If applicable, is the statistical analysis and its interpretation appropriate?

Yes

Are all the source data underlying the results available to ensure full reproducibility?

Yes

Is the study design appropriate and is the work technically sound?

Yes

Are the conclusions drawn adequately supported by the results?

Yes

Are sufficient details of methods and analysis provided to allow replication by others?

Yes

Reviewer Expertise:

Animal health economics

I confirm that I have read this submission and believe that I have an appropriate level of expertise to confirm that it is of an acceptable scientific standard.

# Peer review: Reviewer response for version 1

**DOI:** 10.21956/openresafrica.14545.r29861

**Published:** 2023-07-26

**Authors:** Liam Morrison

**Affiliations:** 1 The University of Edinburgh, Edinburgh, Scotland, UK

The article by Serem et al describes the results of a structured questionnaire approach to assess the knowledge, perception and practices of livestock trypanosomiasis and the tsetse vector in a cohort of farmers in areas neighbouring the Arabuko-Sokoke Forest Reserve, Kenya. Importantly, sites for data collection and participants were selected using randomisation methods, and were assessed for their knowledge of tsetse fly recognition, role in disease transmission, and clinical presentation and control practices in livestock. The data indicated correlation of knowledge with level of education, age and employment status of the respondents. The study was well designed, the data presented appropriately and thoroughly, and there is good placing of the study findings in wider context of comparable literature. As a very minor point, *T. evansi *and *T. equiperdum* are referred to as distinct species in the introduction; it is widely accepted now that these parasites are petite mutants of *T. brucei*, and it would perhaps be more appropriate to refer to them as *T. b. evansi* and *T. b. equiperdum*. Overall the study was well designed and described, and contributes important knowledge to understanding that can inform how to better target information on controlling tsetse and trypanosomiasis to populations in disease-endemic areas.

Is the work clearly and accurately presented and does it cite the current literature?

Yes

If applicable, is the statistical analysis and its interpretation appropriate?

Yes

Are all the source data underlying the results available to ensure full reproducibility?

Yes

Is the study design appropriate and is the work technically sound?

Yes

Are the conclusions drawn adequately supported by the results?

Yes

Are sufficient details of methods and analysis provided to allow replication by others?

Yes

Reviewer Expertise:

My research focus is livestock trypanosomiasis; however, I am not an epidemiologist, and so am only able to comment superficially on the approach and methodology taken.

I confirm that I have read this submission and believe that I have an appropriate level of expertise to confirm that it is of an acceptable scientific standard.
